# The Association of *DRD2* with Insight Problem Solving

**DOI:** 10.3389/fpsyg.2016.01865

**Published:** 2016-11-24

**Authors:** Shun Zhang, Jinghuan Zhang

**Affiliations:** Department of Psychology, Shandong Normal UniversityJinan, China

**Keywords:** creativity, insight, insight problem solving, dopamine, *DRD2*

## Abstract

Although the insight phenomenon has attracted great attention from psychologists, it is still largely unknown whether its variation in well-functioning human adults has a genetic basis. Several lines of evidence suggest that genes involved in dopamine (DA) transmission might be potential candidates. The present study explored for the first time the association of dopamine D2 receptor gene (*DRD2*) with insight problem solving. Fifteen single-nucleotide polymorphisms (SNPs) covering *DRD2* were genotyped in 425 unrelated healthy Chinese undergraduates, and were further tested for association with insight problem solving. Both single SNP and haplotype analysis revealed several associations of *DRD2* SNPs and haplotypes with insight problem solving. In conclusion, the present study provides the first evidence for the involvement of *DRD2* in insight problem solving, future studies are necessary to validate these findings.

## Introduction

Insight refers to a sudden understanding of a problem or a situation that aids in solving the problem (Ohlsson, 1992; Sternberg and Davidson, 1995). It is widely believed to involve a cognitive reorganization or reconstructing of the elements of a problem or situation, which can dramatically changes how a problem or situation is represented. Insight is of great importance for human development, since it has been considered to be the key process that underlies many important technical and scientific innovations (Nickles, 1978; Gruber, 1979). And many psychologists considered the insight ability as a distinctive characteristic of creative individuals (Sternberg and Davidson, 1983).

The history of insight research can be traced back to the early studies of Gestalt psychologists (e.g., Kohler, 1925). Since then, by using the behavioral methods, great efforts have been made to reveal the cognitive mechanism of insight (Chu and MacGregor, 2011). Recently, benefitting from the development of cognitive neuroscience techniques, insight research has entered a new era. By using electroencephalography (EEG), event-related potentials (ERPs) and functional magnetic resonance imaging (fMRI), a series of studies have identified numerous candidate brain regions (e.g., prefrontal cortex, cingulate cortex, hippocampus, and superior temporal gyrus) that might be involved in insight (Kounios and Beeman, 2009, 2014; Chu and MacGregor, 2011). These findings have definitely led to great progress in our understanding of the insight phenomenon; however, it also should be kept in mind that, there are still many questions remaining to be explored, one of which is whether individual differences in insightfulness in well-functioning human adults has a genetic basis.

Fortunately, recent advances in molecular genetics have permitted direct testing of hypotheses regarding the genetic basis of individual differences, and psychologists now have begun to explore the genetic basis of insight. Since findings from cognitive neuroscience studies generally support the involvement of dopamine (DA)-related brain regions, such as prefrontal cortex, anterior cingulate cortex and hippocampus, in the cognitive processes of insight (e.g., Luo and Niki, 2003; Jung-Beeman et al., 2004; Kounios et al., 2006; Anderson et al., 2009; Aziz-Zadeh et al., 2009; Qiu et al., 2010), genes involved in DA transmission have been of particular interest to explain individual differences in insight problem solving ability. Jiang et al. (2015) first investigated the association of DA-related catechol-O-methyltransferase gene (*COMT*) with insight problem solving, and demonstrated preliminary evidence for the effect of *COMT* on insight problem solving ability. As an initial attempt, this study does provide important insight into the roles of DA-related genes in the neural correlates of insight; however, it should also be noted that, the regulation of DA transmission is a complex network involving multiple genes, the roles of other crucial DA-related genes, such as dopamine D2 receptor gene (*DRD2*), have not been explored.

The *DRD2* gene is located on chromosome 11q22-23. The DA receptor encodes by this gene plays an important role in mediating synaptic DA signaling. Genetic variants of *DRD2* have been repeatedly implicated in insight-related cognitive abilities, such as attention, working memory and cognitive control (e.g., Rodriguez-Jimenez et al., 2006; Zhang et al., 2007; Bertolino et al., 2010; Colzato et al., 2010, 2011; Nymberg et al., 2014; Blasi et al., 2015). More importantly, recent studies have demonstrated that genetic variants of *DRD2* are associated with individual differences in divergent thinking ability (Reuter et al., 2006; Runco et al., 2011; Murphy et al., 2013; Zhang et al., 2014a,b; Takeuchi et al., 2015), which is another crucial component of creativity. Based on this evidence, it is reasonable to expect that *DRD2* may also play an important role in insight problem solving. Therefore, to elucidate the role of *DRD2* in insight, the present study was designed to comprehensively explore the associations of *DRD2* genetic variants with insight problem solving.

## Methods

### Participants and procedure

Four hundred twenty-five unrelated Chinese college students (99 males and 326 females, mean age = 18.92 years old, SD = 0.84) were recruited from Shandong Normal University. All participants were of Han Chinese origin without self-reported history of neurological and psychiatric disorder. This study was approved by the Shandong Normal University's Institutional Review Board and written informed consent was obtained from all participants after a full description and explanation of the study. Participants first completed the psychometric tests, and then their venous blood samples (2.5 milliliters for each participant) were collected by a professional medical assistant.

### Single-nucleotide polymorphism (SNP) selection

To capture most common polymorphisms in *DRD2*, seven tag SNPs (rs4938019, rs4245148, rs4648319, rs4436578, rs7122246, rs1076560, and rs6279) were first selected from HapMap (http://hapmap.ncbi.nlm.nih.gov) genotype data for the Han Chinese population in Beijing (CHB) (Data Rel 27 Phase II + III, Feb09, on National Center for Biotechnology Information B36 assembly, dbSNP b126) by applying the Tagger program as implemented in Haploview (Version 4.2) software (Barrett et al., 2005) with the following criteria: pairwise tagging only, *r*^2^ > 0.80 and minor allele frequency (MAF) > 5%. The seven selected tag SNP captured 59 out of 66 (89%) common alleles (MAF > 5%) of the genomic region of *DRD2* (chr11: 112785528.112851091, based on National Center for Biotechnology Information Genome Build 36.3), with a mean maximal *r*^2^ = 0.95. In addition, eight putative functional SNPs (rs1799978, rs1799732, rs4648317, rs2283265, rs6277, rs6276 and rs6278, and rs1800497) were also genotyped. Table 1 summarizes the final set of genotyped SNPs.

**Table 1:** **Characteristics of the Genotyped SNPs**.

**SNP^a^**	**Position^b^**	**Location**	**Allele (minor/major)**	**MAF (%)**	**HWE *p***
rs1799978	112851561	5′Promoter region	G/A	19.2	0.277
rs1799732	112851462:112851463	5′Promoter region	Del/C	10.5	0.439
rs4938019	112846601	Intron 1	C/T	38.9	0.683
rs4648317	112836742	Intron 1	T/C	40.4	0.547
rs4245148	112825629	Intron 1	T/C	14.4	0.233
rs4648319	112819573	Intron 1	T/C	36.5	0.175
rs4436578	112811975	Intron 1	C/T	42.9	0.921
rs7122246	112809667	Intron 1	A/G	5.3	0.328
rs2283265	112790746	Intron 5	T/G	45.1	0.493
rs1076560	112788898	Intron 6	A/C	44.5	0.695
rs6277	112788669	Exon 7	T/C	5.5	0.369
rs6276	112786607	3′UTR	A/G	48.0	0.497
rs6279	112786283	3′UTR	G/C	47.9	0.560
rs6278	112785934	3′UTR	T/G	42.6	1.00
rs1800497	112776038	3′ flanking region	T/C	42.6	0.921

*HWE, Hardy–Weinberg equilibrium; MAF, minor allele frequency; UTR, untranslated region*.

a *SNPs are listed down the column in sequential order from the 5′ end to the 3′ end of the sense strand of DRD2*.

b *Physical position is based on NCBI Genome Build 36.3*.

### Genotyping

Genomic DNA for each participant was extracted from peripheral venous blood samples using the QIAamp DNA Mini Kit (Qiagen, Valencia, CA, USA). Genotyping for all SNPs was performed at the Beijing Genomics Institute-Shenzhen (BGI-Shenzhen, Shenzhen, China) by using the Sequenom® MassARRAY® iPLEX system (Sequenom, San Diego, CA, USA) according to the manufacturer's instructions. Forward, reverse and extension primers were designed using the MassARRAY Assay Design (Version 3.0) software. For quality control, 5% random DNA samples were genotyped twice for each SNP to calculate genotyping error. The genotyping accuracy was 100%.

### Insight problems

Ten classic insight problems (five verbal problems and five spatial problems) selected from previous studies were used in the present study (Ormerod et al., 2002; Dow and Mayer, 2004). All of these problems could be determined as “pure” insight problems since they all necessarily require a reconstructing process for their solution (Weisberg, 1995). Mathematical insight problems were not selected because they could be solved mathematically instead of through insight. Example of verbal problems: “Lan and Hong were born on the same day of the same month of the same year to the same mother and the same father—yet they are not twins. How is that possible?” Example of spatial problems: “How can you arrange 6 identical pencils in such as way as to form 4 identical triangles whose side areas are all equal, without modifying the pencils in any way?” Problem presentation always alternated between problem types, and participants were given 2 min to solve each problem. After the test, participants were instructed to report whether they had previously knew the problems and the solutions before (the average number of familiar problems was 0.34, SD = 0.85), and performance scores were calculated as percentage correct on unfamiliar problems.

### Statistical analysis

Hardy-Weinberg equilibrium was tested by Fisher's exact test using Plink v1.07 software (Purcell et al., 2007). Single SNP analysis under the additive genetic model was performed using linear regression in Plink. The additive genetic model codes the SNP genotype as the number of minor alleles (0, 1, 2). Pair-wise linkage disequilibrium (LD) and haplotype blocks were assessed by Haploview. Association analysis for the indentified haplotype blocks was performed using linear regression in Plink. Haplotypes with estimated frequency <5% were excluded from the analysis. For single SNP and haplotype analysis, both empirical point-wise *p* values (*p*_*emp*1_) and multiple testing corrected *p* values (*p*_*emp*2_) were obtained by using the maxT permutation procedure implemented within Plink with 10,000 permutations. The advantage of using permutation test to correct for multiple testing is that it incorporates the correlation between phenotypes and/or between genotypes and is therefore less conservative than Bonferroni correction in the context of the present study (the 15 SNPs were in linkage disequilibrium, and the set of tests were not independent). For single SNP analysis, the corrected empirical *p* values accounted for the total number of SNPs, while the corrected empirical *p* values for haplotype analysis accounted for the total number of haplotypes. In both cases, *p*-values of <0.05 were considered as significant.

## Results

### Descriptive statistics

The average accuracies for total, verbal and spatial insight problems were 27.5% (*SD* = 0.19), 24.6% (*SD* = 0.23), and 30.6% (*SD* = 0.24). The correlation between verbal and spatial problem solving scores was 0.32 (*p* < 0.01). No significant effect of age and gender was observed.

MAFs and the results of Hardy–Weinberg equilibrium tests are shown in Table 1. All 15 SNPs were polymorphic with MAF > 5%, and no significant deviation from Hardy–Weinberg equilibrium was observed.

### Single SNP and haplotype analysis

Table 2 summarizes the results of single SNP analysis. In particular, seven SNPs (rs1799732, rs2283265, rs1076560, rs6276, rs6279, rs6278, and rs1800497) showed associations with total and spatial insight problem solving. No association between SNPs and verbal insight problem solving was observed. After correcting for multiple testing, only the significant associations of rs1800497 and rs6278 with spatial insight problem solving remained.

**Table 2:** **Summary results of significant SNPs associated with insight problem solving**.

**SNP**	**Total insight problem solving**						**Spatial insight problem solving**					
	***B***	***SE***	***t***	***R^2^***	***p_*emp1*_***	***p_*emp2*_***	***B***	***SE***	***t***	***R^2^***	***p_*emp1*_***	***p_*emp2*_***
rs1799732	−0.231	0.110	−2.11	0.010	0.038	0.239	−0.258	0.110	−2.35	0.013	0.020	0.142
rs2283265	0.172	0.067	2.55	0.015	0.011	0.087	0.177	0.067	2.64	0.016	0.008	0.067
rs1076560	0.170	0.068	2.50	0.015	0.011	0.098	0.179	0.068	2.64	0.016	0.008	0.067
rs6276	0.163	0.070	2.34	0.013	0.019	0.147	0.170	0.070	2.44	0.014	0.014	0.116
rs6279	0.164	0.069	2.37	0.013	0.018	0.141	0.171	0.069	2.46	0.014	0.013	0.108
rs6278	0.188	0.069	2.73	0.017	0.006	0.055	0.199	0.069	2.89	0.019	0.003	0.034
rs1800497	0.190	0.069	2.75	0.018	0.005	0.053	0.201	0.069	2.90	0.020	0.004	0.033

*Insight problem solving was analyzed for association by linear regression under the additive genetic model. Empirical point-wise p-values (p_emp1_) and multiple testing corrected p-values (p_emp2_) were obtained by 10,000 permutations. Only significant results are shown*.

**Figure 1:**
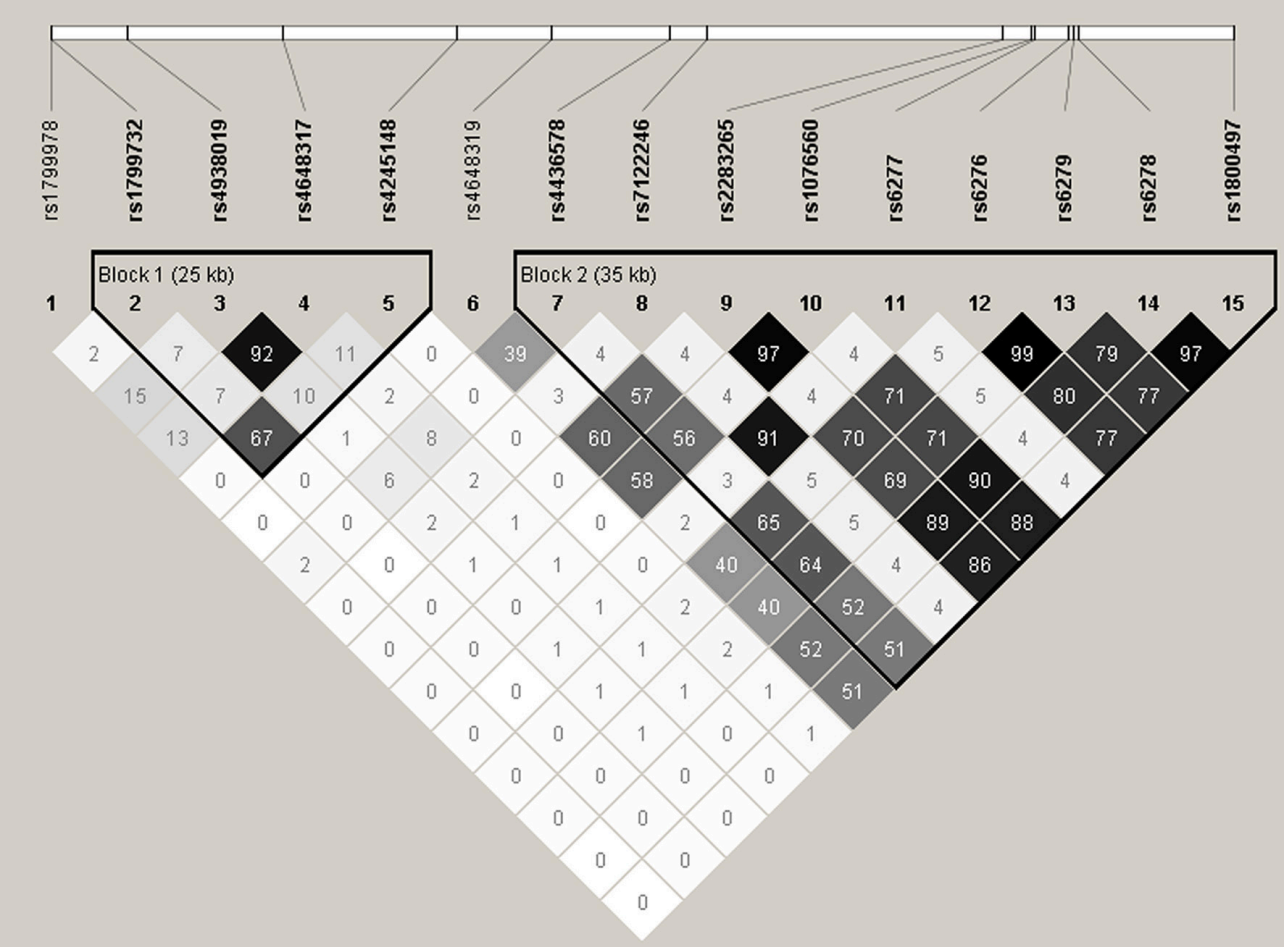
**Linkage disequilibrium (LD) pattern of the 15 SNPs analyzed in the present study**. Numbers in squares designate the degree of LD (*r*^2^) between any two SNPs. LD blocks were defined using the algorithm of Gabriel et al. (2002).

The LD patterns of the genotyped SNPs are shown in Figure 1. There was moderate to strong LD between a number of SNPs, with the strongest LD observed for rs2283265 and rs1076560 (*r*^2^ = 0.97), rs6276 and rs6279 (*r*^2^ = 0.99) as well as rs6278 and rs1800497 (*r*^2^ = 0.97). Two LD blocks were constructed using the algorithm of Gabriel et al. (2002). Block 1 was composed of rs1799732, rs4938019, rs4648317 and rs4245148, and Block 2 was composed of rs4436578, rs7122246, rs2283265, rs1076560, rs6277, rs6276, rs6279, rs6278, and rs1800497. Table 3 summarizes the frequencies of the identified common haplotypes (four from Block 1 and five from Block 2, with frequencies > 5%) and the results of haplotype analysis. For Block 1, although the global test did not reveal any association, the ADTC haplotype (rs1799732-rs4938019-rs4648317-rs4245148) was found to be associated with total and spatial insight problem solving. Block 2 showed associations with total and spatial insight problem solving, with the CTGGCCGCGC haplotype (rs4436578-rs7122246-rs2283265-rs1076560-rs6277-rs6276-rs6279-rs6278-rs1800497) associated with total insight problem solving, and the TTGTACAGTT haplotype associated with spatial insight problem solving. After correcting for multiple testing, only the significant globe association of Block 2 with spatial insight problem solving remained.

**Table 3:** **Associations of haplotypes with total and spatial insight problem solving**.

	**Haplotype**	**Total insight problem solving**						**Spatial insight problem solving**				
		**Frequencies (%)**	***B***	***t***	***R^2^***	***p_*emp1*_***	***p_*emp2*_***	***B***	***t***	***R^2^***	***p_*emp1*_***	***p_*emp2*_***
Block 1^a^	ACCT	39.2	0.022	0.091	0.000	0.755	1.00	0.002	0.00	0.000	0.976	1.00
	ACTC	31.3	0.115	2.48	0.006	0.115	0.614	0.094	1.65	0.004	0.206	0.820
	GCTC	17.9	−0.024	0.075	0.000	0.781	1.00	0.048	0.303	0.001	0.576	0.999
	ADTC	9.9	−0.231	4.45	0.010	**0.038**	0.257	−0.258	5.54	0.013	**0.020**	0.147
	Rare haplotypes	1.7										
	Global test					0.118	0.217				0.114	0.213
Block 2^b^	CCGGCCGCGC	41.8	−0.071	1.04	0.002	0.306	0.937	−0.054	0.598	0.001	0.453	0.989
	TTGTACAGTT	32.1	0.133	3.54	0.008	0.059	0.388	0.146	4.30	0.010	**0.039**	0.275
	CTGTACAGTT	8.5	0.183	2.46	0.006	0.117	0.617	0.196	2.80	0.007	0.092	0.543
	CTGGCCGCGC	6.6	−0.286	4.17	0.010	**0.043**	0.293	−0.242	2.97	0.007	0.085	0.506
	CTAGCTAGGC	5.4	−0.127	0.681	0.002	0.407	0.983	−0.182	1.41	0.003	0.239	0.875
	Rare haplotypes	5.7										
	Global test					**0.034**	0.064				**0.025**	**0.047**

*Haplotype frequencies were estimated using the expectation-maximization (EM) algorithm in Plink and linear regression was used to estimate haplotype-specific effect. An omnibus test was employed to obtain a global p value for the haplotype block. Empirical point-wise p-values (p_emp1_) and multiple testing corrected p-values (p_emp2_) were obtained by 10,000 permutations. Rare haplotypes (estimated frequency <5%) were excluded from the analysis*.

a *The order of SNPs in block 1 was rs1799732, rs4938019, rs4648317, and rs4245418*.

b *The order of SNPs in block 2 was rs4436578, rs7122246, rs2283265, rs1076560, rs6277, rs6276, rs6279, rs6278 and rs1800497. The significance p-values (<0.05) are indicated in bold*.

## Discussion

By examining tag SNPs and putative functional SNPs, the present study systematically explored the association of *DRD2* with insight problem solving.

Of the 15 genotyped SNPs, rs1800497 and rs6278 showed the strongest evidence for the association with insight problem solving. After correcting for multiple testing, these two SNPs were found to be associated with spatial insight problem solving. Rs1800497, also known as the Taq1A polymorphism, located 10 kb downstream from *DRD2*. This variant was historically regarded as a *DRD2* functional variant, and the A1 allele (T allele) has been associated with regulation of the functions of DRD2 receptor by reducing the densities and binding affinity (Noble et al., 1991; Thompson et al., 1997; Pohjalainen et al., 1998; Jönsson et al., 1999; Gluskin and Mickey, 2016). Furthermore, this variant has been repeatedly implicated in insight-related cognitive abilities, such as working memory and divergent thinking (Reuter et al., 2006; Runco et al., 2011; Söderqvist et al., 2013; Nymberg et al., 2014; Zhang et al., 2014b; Takeuchi et al., 2015). Thus, it is reasonable to speculate that, by modulating *DRD2* expression and DA transmission, rs1800497 may affect cognitive abilities that contribute to solving insight problems, and therefore leads to individual difference in insight problem solving. However, one potential deficiency in this explanation is that, although rs1800497 is associated with regulating *DRD2* expression, the exact biological mechanism by which rs1800497 exerts its affect on *DRD2* expression remains to be addressed. Since rs1800497 resides 10 kb downstream from *DRD2*, it is also possible that rs1800497 may actually act as a proxy marker in LD with other functional variants within *DRD2*. This coincides with the finding that the same associations were also observed for rs6278, which is located in the 3′UTR region of *DRD2* and was in nearly complete LD with rs1800497 (*r*^2^ = 0.97). Although the biochemical effect of rs6278 has not been well established, it has been proposed that 3′UTR SNPs may affect mRNA expression by abolishing or creating microRNAs target binding sites in the 3′UTR region (Saunders et al., 2007). It is possible that rs6278 plays a role in regulating *DRD2* expression. Future functional studies are needed to test this hypothesis.

Beside these possibilities, recent studies also suggest another potential mechanism by which rs1800497 may exert its effect on DA transmission and insight problem solving. Although historically regarded as a *DRD2* variant, rs1800497 was recently identified to be a functional coding variant in the ankyrin repeat and kinase domain containing 1 gene (*ANKK1*) located at approximately 10 kb downstream of *DRD2* (Neville et al., 2004). The product of *ANKK1* is a serine/threonine kinase involved in signal transduction, and rs1800497 results in a Glu713Lys substitution in the putative binding domain of *ANKK1* and may alter substrate-binding specificity of *ANKK1* (Neville et al., 2004). It has been suggested that *ANKK1* may affect DA transmission by modulating the phosphorylation of amino acid residues within key proteins (e.g., DA transporters) of DA system (Munafò et al., 2007). Thus, by directly affecting the function of *ANKK1*, rs1800497 may indirectly regulate the activity of DA transporters and DA transmission, which would in turn lead to individual differences in insight-related cognitive abilities and insight problem solving ability. If this mechanism is valid, then the observed association of rs6278 would be due to its strong LD with rs1800497. Nevertheless, this mechanism is also highly speculative, and needs to be verified by future studies.

In addition to rs1800497 and rs6278, the present study also indentified five *DRD2* SNPs (rs1799732, rs2283265 rs1076560, rs6276 and rs6279) nominally associated with total and spatial insight problem solving. Rs1799732 (also referred to as −141C Ins/Del) is a cytosine (C) insertion/deletion polymorphsim located in the 5′ promotor region of *DRD2*. This variant has been demonstrated to be a functional polymorphism, which could putatively alter *DRD2* expression *in vitro* (Arinami et al., 1997) and affect *DRD2* receptor binding in striatum (Arinami et al., 1997; Jönsson et al., 1999). So, by directly affecting *DRD2* expression and DA transmission, rs1799732 might affect insight problem solving. However, it is also possible that rs1799732 might be in high LD with other unidentified causative variants. Unlike rs1799732, the other four SNPs (rs2283265, rs1076560, rs6276, and rs6279) were in moderate or strong LD with rs1800497 and rs6278. Since the association signal from rs1800497 and rs6278 is much stronger than these four SNPs, it is possible that the nominal associations of these four SNPs might be due to their LD with rs1800497 and rs6278. However, as for rs2283265 and rs1076560, it is also possible that they may actually play a role in insight problem solving. Previous functional studies have indicated that, by affecting the relative expression of the two DRD2 receptors isoforms, the D2 long isoform (D2L) and the D2 short isoform (D2S), rs2283265 and rs1076560 may modulate striatal and prefrontal activity during cognitive processes (Zhang et al., 2007; Bertolino et al., 2010). Thus, rs2283265 and rs1076560 may indeed affect insight problem solving; the lack of association after correcting for multiple testing might be partly due to the relatively small sample size. Future studies with larger sample size are warranted to draw a definite conclusion.

Because of the strong LD between genotyped SNPs, haplotype analysis was also performed. However, compared with single SNP analysis, haplotype analysis did not further improve the strength of the associations. Moreover, the genetic effects were similar in gender-specific (males and females) analyses and there was no significant interaction between gender and SNPs and haplotypes (data not shown).

It is intriguing to note that the genetic associations revealed in the present study may also provide supporting evidence for the domain-specific theory of insight problem solving. According to the domain-specific theory, rather than a unitary category of problems that require the same general problem solving skills and cognitive abilities, insight problems are a collection of distinct types of problems that requiring different kinds of problem solving skills and cognitive abilities (Dow and Mayer, 2004). Previous behavioral studies generally supported the domain-specific theory. For example, Dow and Mayer (2004) found that training students to learn to solve spatial insight problems only facilitated solving spatial insight problems, but not other types of insight problems. Gilhooly and Murphy (2005) found that individual differences in vocabulary were associated with better verbal insight problem solving, while differences in spatial flexibility were associated with better spatial insight problem solving. In the present study, association analysis was performed for both verbal and spatial insight problem solving; however, it was found that the identified genetic variants were only associated with spatial insight problem solving, but not verbal insight problem solving. This result implicates that the underlying genetic basis of verbal and spatial insight problem solving might be different; *DRD2*-related genetic variants may uniquely contribute to spatial insight problem solving. This is consistent with our recent finding that rs1800497 and rs6278 were only associated with figural divergent thinking flexibility, but not verbal divergent thinking flexibility (Zhang et al., 2014b). Thus, from genetic and biological perspective, the present study may provide additional evidence for domain-specific theory of insight problem solving.

The present study also has several limitations. First, the participants of the present study were only Han Chinese and the sample size is relatively small. Since both genetic backgrounds (e.g., allele frequencies, LD patterns) and environmental backgrounds vary for different ethnic populations, the generalization of these findings to other populations is limited. Future replication studies in other ethnic populations using larger sample size are warranted to confirm these findings. Second, only classic verbal and spatial insight problems were used in the present study and the number of problems was relatively small. Future studies should further examine whether *DRD2* is similarly related to other measures of insight, such as Matchstick Arithmetic (Knoblich et al., 1999), Compound Remote Associates (CRAs) (Bowden and Beeman, 1998; Bowden and Jung-Beeman, 2003), and Rebus Puzzles (MacGregor and Cunningham, 2008). And the Rebus Puzzles are of particular interest, since this task combines both verbal and spatial cues. Third, the present study only provides preliminary explanations for the observed associations of *DRD2* variants with insight problem solving, all such possibilities remain highly speculative and need to be further refined. Thus, future studies combing both genetic analysis and careful psychological analysis are required to clarify the exact psychological underlying mechanisms by which *DRD2* may affect insight problem solving. Fourth, other crucial genes involved in DA transmission, such as *COMT*, dopamine D4 receptor gene (*DRD4*) and dopamine transporter gene (*DAT1*), were not examined in the present study. Since the regulation of DA transmission is a complex network involving multiple genes, *DRD2* may interact with these genes to affect insight problem solving. Our previous study has shown that *DRD2* may interact with *COMT* to affect divergent thinking flexibility (Zhang et al., 2014a), which is closely related to insight problem solving (Deyoung et al., 2008). And Zabelina et al. (2016) recently also reported that divergent thinking and creative achievement could be predicted by the interaction between *COMT* and *DAT1*.

In conclusion, by systematically exploring the association of *DRD2* with both verbal and spatial insight problem solving, the present study provides the first evidence for the involvement of *DRD2* in insight problem solving. Although needing to be further verified, findings from this exploratory study may provide important and useful information to elucidate the potential genetic effect of *DRD2* on insight problem solving, which may lead to a better understanding of the underlying genetic architectures of insight phenomena.

## Ethics statement

This study was approved by the Shandong Normal University's Institutional Review Board. Written informed consent was obtained from all participants after a full description and explanation of the study. No vulnerable population was involved in the present study.

## Author contributions

SZ, JZ were involved in the conception and design of the work. SZ collected and analyzed the data. SZ, JZ contributed in writing the main manuscript text.

## Funding

This research was supported by National Natural Science Foundation of China (31470999), Natural Science Foundation of Shandong Province, China (ZR2014CQ017), MOE (Ministry of Education in China) Project of Humanities and Social Sciences (16YJC190030), “Tese Mingxiao Zhiliang Gongcheng” project of Shandong Normal University of China.

### Conflict of interest statement

The authors declare that the research was conducted in the absence of any commercial or financial relationships that could be construed as a potential conflict of interest.
